# Romiplostim and Eltrombopag in Immune Thrombocytopenia as a Second-Line Treatment

**DOI:** 10.7759/cureus.9920

**Published:** 2020-08-21

**Authors:** Erjola Bidika, Hafsa Fayyaz, Marina Salib, Areeba N Memon, Asavari S Gowda, Bhavana Rallabhandi, Ivan Cancarevic

**Affiliations:** 1 Internal Medicine, California Institute of Behavorial Neurosciences & Psychology, Fairfield, USA; 2 Internal Medicine, California Institute of Behavioral Neurosciences & Psychology, Fairfield, USA; 3 Medicine, California Institute of Behavioral Neurosciences & Psychology, Fairfield, USA; 4 Neurology, California Institute of Behavioral Neurosciences & Psychology, Fairfield, USA

**Keywords:** romiplostim, eltrombopag, immune thrombocytopenia, platelets, thrombopoietin, thrombopoietin receptor agonists, itp

## Abstract

Immune thrombocytopenia (ITP) is an autoimmune disorder characterized by platelet count less than 100×10^9^/L and an increased risk of bleeding. The risk of bleeding increases in proportion with the degree of thrombocytopenia. Although several medications are used for primary thrombocytopenia treatment, refractoriness remains a concern. Romiplostim and eltrombopag, two relatively new drugs, have been shown to be successful in ITP treatment after standard treatment failure. The current guidelines recommend their use as a second-line treatment. In this article, we have tried to compare which of these two medications is the best option considering clinical effectiveness, cost-effectiveness, adverse effects, and the possibility of switching between them in case of ineffectiveness. The studies used in this article were found in the PubMed database. All the studies are limited to adults. Based on these studies, both medications seem to be a largely effective, safe option. Romiplostim appears to have slightly fewer adverse effects and higher costs. Switching between thrombopoietin receptor agonists (TRAs) is a successful way to overcome adverse effects and inadequacy according to the currently available literature. We believe that more detailed studies are needed to determine which of these drugs should be considered the first choice, to report long term efficacy and adverse effects, and to determine if treatment guidelines can change regarding the use of TRAs as first-line treatment.

## Introduction and background

Immune thrombocytopenia (ITP) is a condition of low platelet count (less than 100×109/L), in which platelets are destroyed by the immune system, with an estimated incidence of 2-4/100000 adults/year [1,2]. In this disorder, platelet production and survival are altered by antibodies, mainly IgGs that attack glycoproteins, GP IIb/IIIa, GP Ib/IX, GP Ia/IIa, and other glycoproteins expressed on megakaryocytes and platelets [3]. Platelets are phagocytosed and destroyed prematurely by the reticuloendothelial system, particularly the spleen. In these patients, the risk of bleeding increases in proportion with the degree of thrombocytopenia [1]. In ITP patients, long term risks, morbidity, and mortality rate RR 2.3 (95% CI, 1.8-3.0) are increased compared to the general population [4]. 

ITP is characterized as primary and secondary, according to the etiology [5]. Primary ITP is defined as isolated thrombocytopenia without other causes [5]. Secondary ITP is defined as any other form of thrombocytopenia except for primary and includes thrombocytopenia secondary to an underlying disease such as hepatitis C infection, systemic lupus erythematosus, or lymphoproliferative disorders [5]. ITP is further categorized as newly diagnosed (<3 months), persistent (3-12 months), and chronic (>12 months) [5]. In children, this disorder is often self-limited, but most adults need treatment [6]. First-line treatment includes IV immunoglobulin, steroids, anti-D-immunoglobulin, and lastly, splenectomy [7]. Rituximab is an alternative treatment in patients who are at increased risk of bleeding after the failure of one of the above treatments [7]. Refractoriness to the previously mentioned therapies led to new treatment strategies. 

Hematologist Endre Kelemen discovered thrombopoietin (TPO) and determined its role in platelet stimulation in 1958. TPO was able to be purified by five laboratories almost simultaneously in 1994 [8,9]. TPO is a hormone consisting of 332 amino acids, synthesized primarily in the liver, which binds to TPO receptors referred to as c-MpL on the megakaryocytic surface [10]. Recombinant human thrombopoietin (rhTPO) and pegylated recombinant human megakaryocyte growth and development factor (PEG‐rhMGDF), were found to increase platelet counts, but their development was discontinued after antibodies that cross-reacted with endogenous TPO were found in healthy volunteers causing paradoxical thrombocytopenia [11,12]. Shortly after this, thrombopoietin receptor agonists (TRAs) were introduced as a promising treatment. TRAs mimic endogenous TPO by activating intracellular signaling pathways such as Janus kinase/signal transducer and activator of transcription (JAK/STAT) and mitogen-activated protein kinase (MAPK) and enhancing platelet count by stimulating megakaryocytes [13,14]. 

In 2008 US Food and Drug Administration (FDA) approved two TRAs, romiplostim and eltrombopag, based on the results of the phase-3 trial published in *The Lancet* [15]. Both drugs are approved in the treatment of ITP in patients who relapse after splenectomy or who have a contraindication to splenectomy, in those who are at increased risk of bleeding or following the failure of at least one other therapy [7]. Romiplostim and eltrombopag have no sequence homology with TPO, thus decreasing the risk of antibody formation [16]. Both drugs bind to the TPO receptor (c-MpL) and stimulate megakaryocyte differentiation and proliferation [16]. Romiplostim is a recombinant, Fc-peptide fusion protein (peptibody) given subcutaneously, while eltrombopag is an orally available drug that binds to the transmembrane region of c-MpL [17]. Recognizing that ITP pathophysiology is defined as increased destruction of platelets and decreased production, the treatment should also not be limited to focusing only on diminishing platelet destruction, but also increasing survival [18]. Therefore, the use of romiplostim and eltrombopag in ITP treatment is supported by promoting megakaryocyte survival and increasing platelet production [18]. In this review article, we will review and compare the effectiveness and side effects of romiplostim and eltrombopag in ITP treatment and the validation of switching between these two drugs, based on previously published studies. 

## Review

Romiplostim and eltrombopag clinical response 

The normal platelet count in the general population ranges between 150 and 400×10^9^/L while in ITP patients treated with TRAs, the main treatment goal is to decrease the risk of bleeding and maintain platelet count between 50×10^9^/L and 150×10^9^/L [18]. However, in patients with a severe disease without bleeding, a platelet count of ⩾20×10^9^/L is tolerable [18]. Several studies have been done to compare the outcomes in patients treated with romiplostim to patients treated with standard treatment or placebo. Kuter et al. studied 157 patients with pretreatment platelet counts 50×10^9^/L or less, who were treated with romiplostim in a 52-week study [19]. They found a significant increase in platelet counts, a lower rate of treatment failure, a lower rate of splenectomy and bleeding, and a significant improvement in life quality [19]. In a 24-week long study Kuter et al. analyzed 83 patients treated with romiplostim from whom 50% splenectomized and with a pretreatment platelet count 30×10^9^/L or less [15]. They reported increased platelet counts in 79% of splenectomized patients and 88% in non-splenectomized patients [15]. They also reported the ability to reduce the use of other ITP medications [15]. Bussel et al. studied 41 patients for six weeks [20]. The platelet counts were reported to be increased to 50×10^9^/L or more [20]. Shirasugi et al. analyzed the outcomes in 22 Japanese patients treated with romiplostim in a 12-week study, with a pretreatment platelet count of 30×10^9^/L or less and 44% of whom had been splenectomized [21]. They reported a platelet response in 95% of the patients and a reduced need for rescue therapy (only in nine percent of patients) [21]. Several studies have been made to assess the effectiveness of eltrombopag. Cheng et al. studied 135 patients from 23 different countries treated with eltrombopag in a three-phase, 24-week study [22]. Pretreatment platelet counts in these patients were 30×10^9^/L or less, and the splenectomy rate 36% [22]. They reported increased platelet counts in 79% of patients and a reduced need for rescue therapy or other ITP medications [22]. Bussel et al. studied 118 patients who had a refractory or relapsed ITP in a six-week study [23]. Their pretreatment platelet counts were less than 30×10^9^/L, and 47% had undergone splenectomy [23]. A significant increase in platelet counts and a decrease in bleeding were reported in 80% of patients treated with eltrombopag by the end of the second week [23]. Yang et al. analyzed in an eight-week study the response of treatment with eltrombopag in 104 Chinese patients [24]. 51.9% of the patients had pretreatment platelet counts less than 15×10^9^/L, and 17.3% had undergone splenectomy [24]. A significant response was reported in 57.7% of patients after six weeks of treatment with eltrombopag [24]. Bussel et al. studied 76 patients from 23 countries in a six-week study [25]. Patients had pretreatment platelet counts less than 30×10^9^/L, and 39% were splenectomized [25]. They reported a significant response with increased platelet counts in 59% of patients treated with eltrombopag [25]. Tomiyama et al. studied 15 Japanese patients in a six-week study, with platelet counts less than 30×10^9^/L and from whom 69% were splenectomized [26]. They reported a response to treatment in 60% of patients, decreased bleeding, and a lower eltrombopag dose to be effective in Japanese patients [26]. The included studies can be found in Table 1.

**Table 1:** Characteristics of Reviewed Studies

Number of patients	Study,year	Country	Splenectomized	Pretreatment platelet counts	Platelet response	Study duration
ROMIPLOSTIM						
157	Kuter et al., 2010 [19]	USA, Europe, Australia	0%	<50×10^9^/L	⩾50×10^9^/L	52 weeks
83	Kuter et al., 2008 [15]	USA, Europe	50%	<30×10^9^/L	⩾50×10^9^/L	24 weeks
41	Bussel et al., 2006 [20]	USA	67%	-	⩾50×10^9^/L	6 weeks
22	Shirasugi et al., 2011 [21]	Japan	44%	<30×10^9^/L	⩾50×10^9^/L	12 weeks
ELTROMBOPAG						
135	Cheng et al., 2011 [22]	23 countries	36%	<30×10^9^/L	⩾50×10^9^/L	24 weeks
118	Bussel et al., 2007 [23]	USA	47%	<30×10^9^/L	⩾50×10^9^/L	6 weeks
104	Yang et al., 2017 [24]	China	17.3%	51.9% of patients < 15×10^9^/L	⩾50×10^9^/L	8 weeks
76	Bussel et al., 2009 [25]	23 countries	39%	<30×10^9^/L	⩾50×10^9^/L	6 weeks
15	Tomiyama et al., 2012 [26]	Japan	69%	<30×10^9^/L	⩾50×10^9^/L	6 weeks

**Figure 1:**
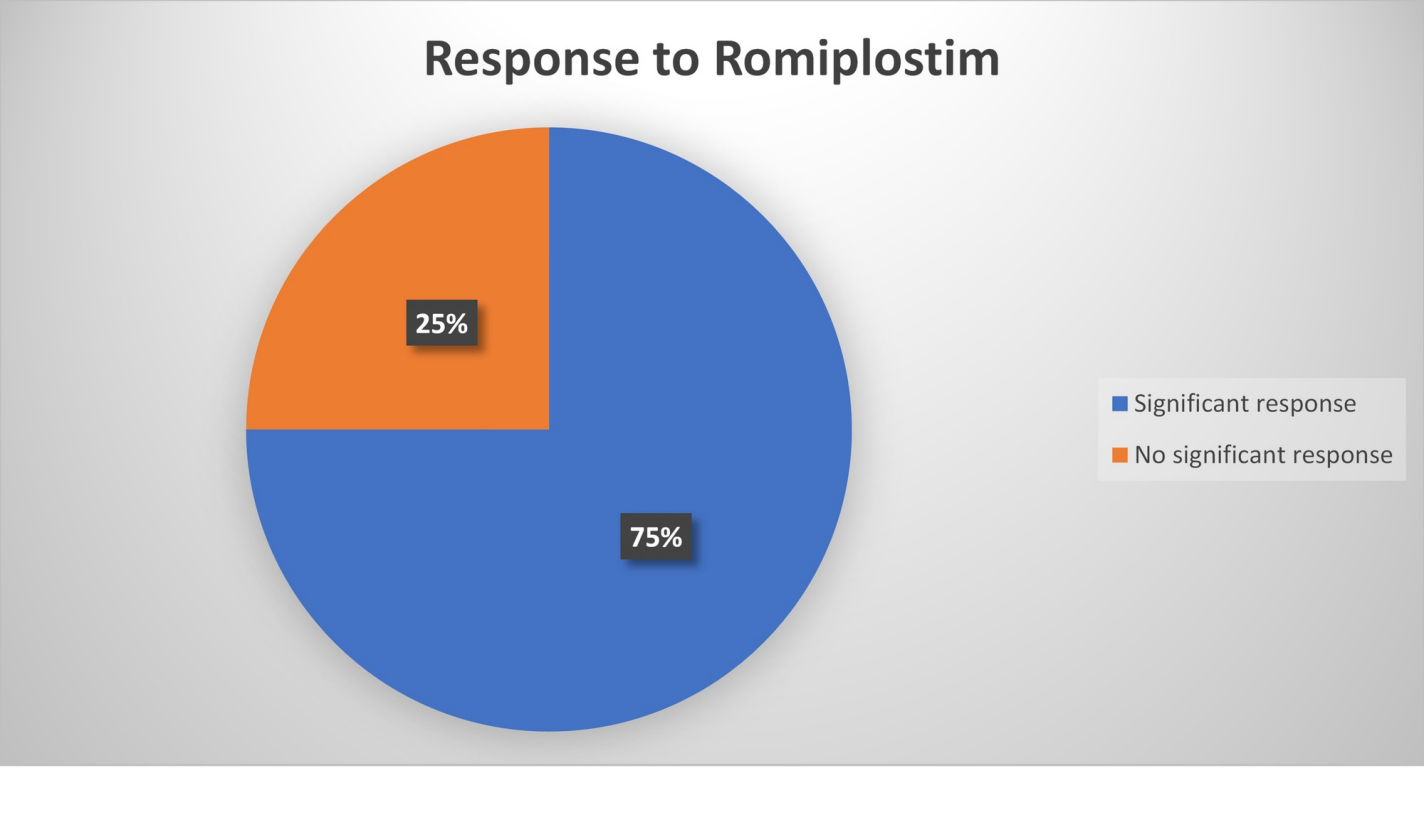
Response to Treatment With Romiplostim

**Figure 2:**
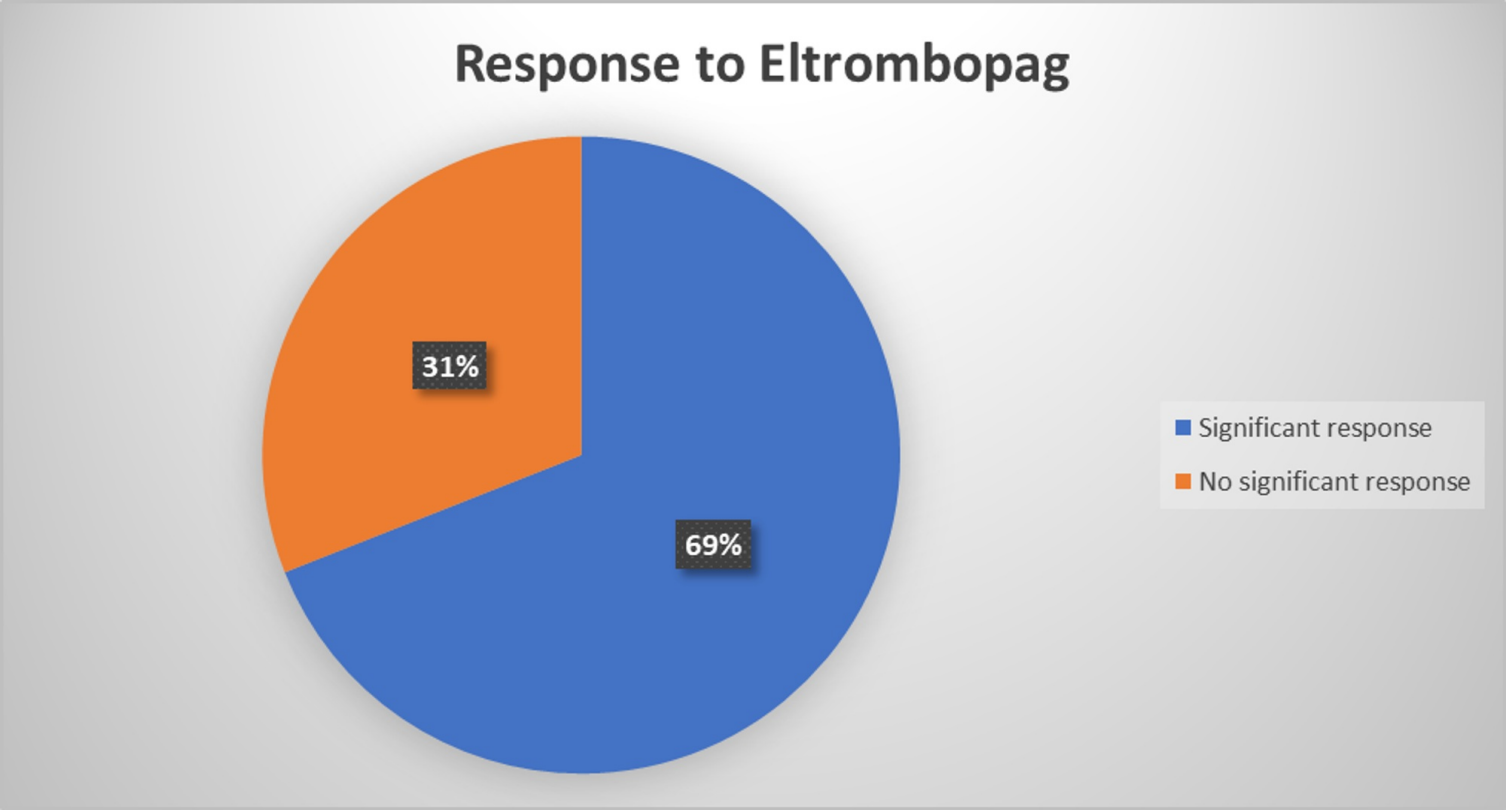
Response to Treatment With Eltrombopag

Overall, 303 patients treated with romiplostim were included in these studies, with an average of 75% showing a significant response in platelet counts. Four of the patients were reported to have post-treatment worsening thrombocytopenia. Three patients reported to have platelet counts exceeding 450×10^9^/L and two patients had extended response after the period of treatment. The number of patients treated with eltrombopag in these studies overall was 448, where 313 of them (69%) responded to the treatment. These findings can be found in Figure 1 and Figure 2. Treatment with both romiplostim and eltrombopag resulted in significantly higher platelet counts response than placebo. The clinical response was higher than that of the other second-line treatment agents. A significant decrease of hemorrhagic episodes was also reported. In some studies, the efficacy of eltrombopag resulted in being dose-dependent. In another study, the starting efficacy dose of eltrombopag was lower in East Asian patients. Considering the high response of ITP patients treated with romiplostim and eltrombopag, these two agents seem to be a precious treatment in refractory cases.

Adverse effects of romiplostim and eltrombopag 

Multiple studies have concluded that romiplostim and eltrombopag are generally well-tolerated medications [19,27,28]. However, some adverse effects have been reported. Shirasugi et al. reported that the most common adverse effects experienced by patients treated with romiplostim were headache, back pain, extremity pain, nasopharyngitis, and peripheral edema [21]. Myalgia, headache, dizziness, insomnia, and arthralgia were also reported in patients treated with romiplostim [19]. One patient reportedly experienced a multiorgan failure, which resolved after romiplostim discontinuation [29]. Cheng et al. found that among 135 patients treated with eltrombopag, three had episodes of thromboembolic events, nine patients had a mild increase of ALT levels, and five patients had an increase in total bilirubin [22]. Among 104 patients treated with eltrombopag, Bussel et al. reported hypokalemia in 11 patients, increased ALT in nine patients, and nasopharyngitis in 11 patients. Only in four patients were severe adverse effects reported [23]. The EXTEND and the RAISE studies reported mostly mild to moderate adverse effects in patients treated with eltrombopag [30]. Fewer serious adverse effects were reported [30]. Mild to moderate adverse effects include nausea, dizziness, headache, and muscle aches [30]. Severe adverse effects include rebound thrombocytopenia, stroke, myocardial infarction, cataracts, and vascular occlusions [30]. Although the EXTEND study demonstrated an increase in alanine aminotransferase (ALT) up to three times the upper limit in 10% of patients, ALT levels returned to normal even when patients continued the treatment or when they discontinued it. Further, this study demonstrated that indirect bilirubin was increased, suggesting that the liver injury was not serious [31].

Kim et al. analyzed 18 patients and reported that seven patients on eltrombopag showed abnormal hepatobiliary laboratory values [31]. However, ALT levels returned to normal after treatment discontinuation [31]. One of the patients showed bone marrow fibrosis after the first year of treatment with eltrombopag [31]. A systematic review and meta-analysis of randomized controlled trials suggested an increased risk of thromboembolic events in the patients treated with TRAs compared with placebo or standard care [32]. Long-term observational studies in patients treated with TRAs suggested that a minority of them developed bone marrow fibrosis [33,34]. However, it was found to be reversible and dose-dependent [33,34]. Moulis et al. concluded that the risk of gastrointestinal adverse effects is higher with eltrombopag. However, hematological adverse effects are higher with romiplostim, and no significant difference is noted between them, according to thrombosis [35]. Loffredo and Violi also reported an increased risk of thromboembolic events in patients treated with eltrombopag and who had a chronic liver disease [36]. 

Although adverse effects have been reported in multiple studies, they seemed to be mild and reversible after drug discontinuation. Most of the adverse effects reported seem not to be a significant concern, but adverse effects such as abnormal liver function tests require continuous monitoring so that the treatment can be modified accordingly. Liver function test abnormalities are mainly seen in patients treated with eltrombopag, and no significant changes are reported in patients on romiplostim. Neither of these two agents is associated with significantly increased rates of severe adverse effects. However, cases of bone marrow fibrosis and thromboembolic events have been reported with both drugs. It is especially important to perform a detailed evaluation of thrombosis risk and whether it is due to the inflammation in ITP itself or due to treatment with TRAs. Overall, long-term treatment with romiplostim and eltrombopag is considered a largely safe and well-tolerated option with romiplostim showing a slight dominance. 

Switching between TRAs 

Lack of effectiveness refers to a lack of platelet count response to prior treatment, or an inability to maintain stable platelet counts [37]. These are reported as the main reasons for therapy switching [37]. Patient preference is another reason, with pre-dominance to eltrombopag switch (32%) compared to romiplostim (22%) [37]. Kuter et al. reported platelet counts to rise from mean 41.0×10^9^/L to 138×10^9^/L with romiplostim and from 43.4×10^9^/L to 120×10^9^/L with eltrombopag [37]. They suggested that changing between TRAs may be appropriate in patients who fail to respond to the initial TRA used [37]. González-Porras et al. reported a 75% response in patients who switched between TRAs [38]. Better outcomes were reported in patients where the reason for switching was adverse effects or patient preference than in ineffectiveness to prior TRA [38]. Even though 16 patients responded to treatment with romiplostim, they requested switching to eltrombopag due to the convenience of taking the drug orally [38]. Depré et al. summarized the outcomes of published studies and reported that in patients who switched to romiplostim due to treatment ineffectiveness, 73% responded positively. All patients who switched to romiplostim due to adverse effects responded positively [29]. Of patients who switched from romiplostim to eltrombopag due to treatment ineffectiveness, 58% responded positively. Of those who switched due to platelet counts fluctuations, 71% responded positively [29]. Of those who switched due to adverse effects, 92% responded positively, and in patients who switched due to patient preference, 100% responded positively [29]. They observed that adverse effects were decreased from 27 to nine in patients who switched from eltrombopag to romiplostim and increased from 12 to 19 in patients who switched from romiplostim to eltrombopag [29]. Several other studies suggest that switching between TRAs is appropriate in case of the ineffectiveness of the prior agent [17,24]. 

The above studies support the switching between TRAs as an effective way to overcome treatment resistance and adverse effects. An increase in platelet response rate was achieved in the majority of cases. Switching due to treatment resistance seems unsuccessful compared to switching due to the adverse effects or platelet level fluctuations. Switching to romiplostim appears to be more effective. On the other hand, switching to eltrombopag appears to be more concerning regarding increased adverse effects. 

Cost-effectiveness 

Considering the high cost of TRAs, research has been done to estimate the overall cost of these drugs. Allen et al. analyzed the cost between patients treated with romiplostim and eltrombopag in England and Wales and concluded that eltrombopag is cost-effective compared to romiplostim. Eltrombopag was found to be £40,261 ($50,501) less expensive in every non-splenectomized ITP patient during their lifetime and £88,904 ($111,516) less expensive in every splenectomized ITP patient [39]. Tremblay et al. analyzed the cost of TRAs for patients treated in the USA [40]. According to this study the overall cost for eltrombopag is approximately $66,560 ($65,998 in splenectomized patients and $67,151 in non-splenectomized patients) and for romiplostim $91,039 ($91,485 in splenectomized patients and $91,455 in non-splenectomized patients) [40]. Fust et al. concluded that romiplostim is cost-effective related to eltrombopag with a slight difference [41]. Al-Samkari reported a romiplostim cost of $2165.34 per 250 µg vial or $4330.68 per 500 µg vial [17]. For eltrombopag, a cost of $182.46 per 12.5 mg or 25 mg tablet, $330.20 per 50 mg tablet, and $495.30 per 75 mg tablet was reported [17]. 

While both drugs have high overall cost, in England, eltrombopag is more cost-effective than romiplostim. In the USA, the studies oppose each other, but based on the cost per eltrombopag tablet and romiplostim vial, we conclude that romiplostim cost is higher.

## Conclusions

ITP treatment after first-line therapy failure remains a challenge for clinicians. The introduction of TRAs romiplostim and eltrombopag was a significant step for refractory ITP treatment. Several studies in the last decade have shown promising results, however long-term efficacy and adverse effects seem unclear. Overall, both drugs have proved to be a reasonable treatment option in increasing platelet count and survival. Adverse effects appear to be mostly mild and reversible with drug discontinuation. Switching between TRAs remains a good option, and both drugs have a high cost. Long term studies would be of significant help in determining long term severe adverse effects and long-term efficacy. Currently, we recommend TRAs use for refractory ITP treatment, especially in the setting of limited options. Further studies should be done to assess TRAs use as a first-line treatment.
